# Accomplishment of instrumental activities of daily living and its relationship with cognitive functions in adults with myotonic dystrophy type 1 childhood phenotype: an exploratory study

**DOI:** 10.1186/s40359-021-00562-1

**Published:** 2021-04-17

**Authors:** Marjolaine Tremblay, Samar Muslemani, Isabelle Côté, Cynthia Gagnon, Julie Fortin, Benjamin Gallais

**Affiliations:** 1 Groupe de Recherche Interdisciplinaire Sur Les Maladies Neuromusculaires (GRIMN), Centre Intégré Universitaire de Santé Et de Services Sociaux du Saguenay-Lac-St-Jean, Québec, Canada; 2 Centre de Recherche Charles-Le-Moyne-Saguenay-Lac-Saint-Jean Sur Les Innovations en Santé, Université de Sherbrooke, Québec, Canada; 3 ÉCOBES - Recherche Et Transfert, Cégep de Jonquière, 2505 rue Saint-Hubert, Jonquière, QC G7X 7X2 Canada

**Keywords:** Myotonic dystrophy, Independent living, Cognition, Instrumental activities of daily living, Participation

## Abstract

**Background:**

The childhood phenotype of myotonic dystrophy type 1 (DM1) involves impaired cognitive functioning starting in infancy, which may compromise later on their ability to carry out instrumental activities of daily living (IADLs) necessary for living independently. The current study aims to document the ability to perform IADLs among adults with the childhood phenotype of DM1 and to explore its links to cognitive functioning.

**Methods:**

This cross-sectional exploratory study was conducted among 11 individuals living with DM1. IADLs related to money management, home management & transportation and health & safety activities were assessed by the Independent Living Scale (ILS). Neuropsychological tests assessed participants’ intellectual abilities and executive functioning. Associations were investigated using Spearman’s rho correlation.

**Results:**

Important difficulties were found in all three categories of IADLs, mostly in money management in which only 2/11 participants were scored as independent. 8/11 participants showed low to very low intellectual functioning and limit to impaired executive functioning. Apathy was also a common feature as 5/11 participants showed clinical level of apathy. A lower IQ was associated with greater difficulty in the home management & transportation subtest of the ILS.

**Conclusions:**

Adults with the childhood phenotype of DM1 demonstrate relative dependence in regard to the following IADLs: money management and home management & transportation. Level of dependence is, at least partially, associated with cognitive impairments. The work relates to results from an exploratory study; thus, studies must be pursued to describe in more details difficulties experienced by this population.

## Background

Myotonic dystrophy type 1 (DM1) is a progressive autosomal dominant disorder. It is the most frequently inherited neuromuscular disease, with an estimated incidence of 5 to 20 per 100,000 individuals [1]. DM1 is a multisystemic disease, meaning that in addition to the muscular involvement (myotonia, muscular wasting and weakness), there are also impairments in cardiac, respiratory, endocrine, ocular, skeletal and central nervous systems (CNS) [2, 3].

DM1 is usually classified into five phenotypes [4, 5]: (1) congenital, (2) childhood, (3) juvenile, (4) adult and (5) late-onset. Most studies have been conducted among the adult and late-onset phenotypes and showed the importance of conducting phenotype-specific studies has they present in different challenges in regard to impairments, activity and participations restrictions [4, 6, 7]. Among them, the childhood phenotype has been scarcely described although being regarded as presenting with a more severe long-term prognosis in relation to independent living. It is characterized by facial weakness, dysarthria, hand muscle myotonia and delayed motor development [8], occurring between 1 and 10 years of age with absence of neonatal problems. In addition, children with the DM1 childhood phenotype usually present with lower IQ levels and cognitive impairments [9–11] such as speech and language delay, learning disability and lower performance in relation to adaptive behaviors, socialization, communication, and daily living tasks [10, 12]. The evolution of cognitive disability has provided conflicting results in individuals with the childhood phenotype; some documented a decline of the general IQ [13, 14] whereas a recent study documented rather stability in cognitive abilities and adaptive behavior over a 7 years period [15]. Those studies have included almost only child or adolescents below 18 years old. Hence, the cognitive profile of persons with the childhood phenotype remains nearly unknown in adult life. More specifically, it is unclear whether the cognitive profile from childhood to adulthood is stable or declines over time and the impact it could have on their ability to live independently.

Independent living has been known to be disrupted among the DM1 population [16, 17]. At the Saguenay Neuromuscular Clinic (Quebec, Canada), precarious situations such as substandard housing, loss of employment, bankruptcy, etc. have been observed among patients by healthcare professionals in clinical follow-up, which have raised important questions regarding their abilities to live independently. A retrospective chart review was thus conducted and identified that several adult patients with the childhood phenotype experienced participation restrictions in relation to independent living, including home management and economic self-sufficiency (Gagnon et al. 2017). However, no prospective study to our knowledge has yet explored independent living among adults living with the childhood phenotype of DM1. Given the cognitive profile and the low compliance in clinical visits in this phenotype, it was essential to conduct an exploratory study to ensure that patients were able to answer our assessment battery within the two visits scheduled.

Independent living is a complex notion and includes the accomplishment of activities of daily living (ADLs), which can be divided in two main categories: basic ADLs (BADLs) and instrumental ADLs (IADLs). BADLS are usually simple self-care tasks aimed to answer basic physical needs, such as brushing teeth, dressing or grooming. IADLs are more complex activities required to participate to domestic and community life, such as cooking, money management, transportation, etc. IADLs are usually the first affected activities with cognitive decline as they require higher neuropsychological processing [18]. It has been demonstrated in the elderly population that memory and other cognitive functions such as executive abilities may result in problems performing IADLs, therefore jeopardizing their ability to live independently [19–21]. Several studies have shown that cognitive impairments may cause problems in IADLs performance among other populations [22–25]. To our knowledge, the link between cognitive functioning and IADLs has never been investigated in the DM1 population, including adults living with the childhood phenotype.

The objectives of this exploratory study were to document performance-based IADLs accomplishment and to explore their links with the cognitive profile, among adults with the childhood phenotype of DM1.

## Methods

### Participants

Participants were recruited between July and October 2016 among patients with a DM1 childhood phenotype followed at the Saguenay Neuromuscular Clinic (Québec, Canada). Inclusion criteria were: (1) having a diagnosis of DM1 confirmed by DNA analysis; (2) being classified as having the childhood phenotype based on Koch’s classification [5]; (3) being aged over 18 years; and (4) being able to provide informed consent. Eleven participants were recruited using a stratified purposive sampling by age groups (18–29; 30–39; 40–49; 50–59) and sex to ensure sample representativeness.

### Data collection

Participants were seen over two half-day sessions. Prior to the first session, all DM1 participants answered a sociodemographic questionnaire over the phone. The outcome measures regarding IADLs were administered by a trained research professional (registered social worker) at the participant's home. Assistance was given when questions arose. Participants were seen at home at the second session to complete the neuropsychological tests’ battery by a trained neuropsychologist.

### Outcome measures

#### Accomplishment of IADLs

IADLs accomplishment was assessed with a performance-based measure and a retrospective chart review. The Independent Living Scale (ILS) is an assessment of adults’ competence in IADLs [26]. It requires the participant to achieve problem solving, to demonstrate knowledge or to perform a task that allows a direct and objective evaluation of the person’s functioning in daily life. The ILS is comprised of five subscales: (1) Memory/Orientation, (2) Social adjustment, (3) Money management, (4) Home and transportation and (5) Health and safety. These scores are added to obtain an overall score reflecting the participant’s ability to function independently (total score). Only the last three categories were considered as IADLs. The other subscales (memory/orientation and social adjustment) were not considered IADLs because they are not concrete activities but are still important in the assessment of independent living. Raw scores are derived into standard scores, which ranged from 20 to 63 for individual categories, and from 55 to 121 for the total score, where a higher score indicates better capacities. The ILS presents excellent inter-rater reliability (K = 0.99) and good test–retest reliability (r = 0.80) with the elderly [26]. There was no published French version of the ILS, so the research team translated test’s instructions prior to its administration following a forward translation method with one translator and two reviewers with a background in occupational therapy and neuropsychology.

A structured review of participants’ health records from the Neuromuscular Clinic (interdisciplinary rehabilitation team) was conducted by a research professional (registered social worker) to gather information about services received by participants regarding IADLs such as cleaning services. A standardized grid was used to collect information according to the type of assistance needed for each IADL category of the ILS: no help, help from family and/or external resources such as home support services.

#### Neuropsychological assessment

Intellectual abilities were assessed by the Wechsler Abbreviated Scale of Intelligence II (WASI-II), a robust test providing estimate of full-IQ scores, as well as Verbal and Performance IQs in only 4 subtests and within 30 min [27]. Executive functioning was assessed using a short cognitive and behavioral battery to assess frontal lobe functions, namely the Frontal Assessment Battery (FAB) [28]. Additionally, level of apathy was assessed with the Lille Rating Apathy Scale (LARS) [29], for its possible impact on IADLs (higher level of apathy may be associated with a reduced level of activity, independently of the level of functional impairment) [30, 31]. A positive score at the LARS indicated greater level of apathy. Finally, levels of state anxiety, used as a control variable, was assessed with the State questionnaire of the State-Trait Anxiety Inventory (STAI) Y-form (STAI-Y) [32].

### Data analysis and interpretation

Descriptive statistics were used to present participants’ sociodemographic data (mean scores and standard deviation). Given the sample size and the objectives of the study, we used cut-off scores to categorize participants according to performance or level of independence reflecting their potential for independent living. For the ILS, standardized cut-off scores are available and were used to classify participants in three distinct categories for each subscale: independent (activity accomplished ≥ 50), semi-independent (40–49) and dependent (≤ 39). For the total score, the classification was independent (activity accomplished ≥ 100); semi-independent (85–99); and dependent (≤ 84), with a maximum score of 121. The standardization process was made from a sample of 590 healthy older adults (age ≥ 65 years old) in the United States population, depending on the living status. Participants’ intelligence scores (WASI-II) were compared with standardized normative data (after age transformation of raw scores), and executive functioning participants’ scores (FAB) were classified as abnormal if < 16, limit if = 16 and normal if > 16. Regarding apathy (LARS), the questionnaire has a cut-off score of –16, indicating clinical apathy. Moreover, a more refined classification of the intensity of apathy is proposed by authors as follows: [− 36;− 22] for non-apathetic and [− 21;− 17], [− 16;− 10], and [− 9;+ 36] for slightly, moderately, or severely apathetic subjects, respectively. To explore the links between the ILS raw subscale scores and total scores of neuropsychological tests (WASI-II, BREF, LARS), Spearman’s rho correlations were performed. Due to the small sample size, non-parametric analyses were used and only correlation coefficients are presented, not the related p-values. Data were analyzed using IBM SPSS Statistics for Windows, Version 25.0 (Armonk, NY: IBM Corp).

## Results

### Sociodemographic data

Demographic and personal characteristics are presented in Table 1. Participants were aged between 18 and 59 years old (mean age of 39.9 years) and 6 out of 11 were women. In the sample, only one participant lived alone. Only one participant had completed high school and was pursuing further education. None of them had held a remunerative employment. Most participants were not experiencing difficulties in indoor and outdoor mobility (9/11 and 7/11 respectively). Additionally, participants presented low to very low levels of anxiety.

**Table 1:** Demographic and personal characteristics of the participants (n = 11)

Characteristics		n (%)
Sex	Male	5 (45)
	Female	6 (55)
Age	18–19	3 (27)
	30–39	3 (27)
	40–49	2 (18)
	50–59	3 (27)
Living environment	Alone	1 (9)
	With partner	4 (36)
	With parent(s)	4 (36)
	With roommate(s)	1 (9)
	Foster home	1 (9)
School education	≤ 12th grade	11 (100)
Indoor mobility	Without help	9 (82)
	Walking aid	2 (18)
Outdoor mobility	Without help	7 (64)
	Walking aid	2 (18)
	Manual wheelchair	2 (18)
Income	Social assistance	7 (64)
	Family support	2 (18)
	Tax credit	1 (9)
	Unknown	1 (9)
Anxiety	Very low	8 (73)
	Low	3 (27)
	Mild	0
	High	0
	Very high	0

Because of rounding, percentages might exceed 100%

### IADLs accomplishment

IADLs accomplishment results are presented in Table 2 and level of assistance for each IADL according to the health records are presented in Table 3. The IADLs with the lowest independence level was money management, followed by home management and transportation. According to the ILS, a large proportion of participants (8/11) were dependent for money management, which is corroborated by health records indicating that 8/11 received help from their family and 1/11 received help from both family and external services. According to the ILS, participants were mostly semi-independent (6/11) in home management and transportation; 8/11 required help from their family and 2/11 from both family and external services. More than half the participants were independent regarding health and safety abilities (6/11), but information gathered from health records indicated that 9/11 received help from their family in this activity. According to the health records, only one participant required no help in all categories.

**Table 2:** Level of independence for each subscale and for the total score of the ILS (n = 11)

Categories	Level of independence	ILS n (%)
Memory/Orientation	Independent	8 (73)
	Semi-independent	2 (18)
	Dependent	1 (9)
Money management	Independent	2 (18)
	Semi-independent	1 (9)
	Dependent	8 (73)
Home management and transportation	Independent	2 (18)
	Semi-independent	6 (55)
	Dependent	3 (27)
Health and safety	Independent	6 (55)
	Semi-independent	1 (9)
	Dependent	4 (36)
Social adjustment	Independent	2 (18)
	Semi-independent	5 (45)
	Dependent	4 (36)
Total scores	Independent	3 (27)
	Semi-independent	4 (36)
	Dependent	4 (36)

Because of rounding, percentages might not add up to 100% exactly

**Table 3:** Number of participants who needed help to accomplish instrumental activities of daily living according to health records (n = 11)

IADL category	Level of assistance	n (%)
Money management	No help	2 (18)
	Family	8 (73)
	Family and external resources	1 (9)
Home management and transportation	No help	1 (9)
	Family	8 (73)
	Family and external resources	2 (18)
Health and safety	No help	2 (18)
	Family	9 (82)
	Family and external resources	0 (0)

Because of rounding, percentages might not add up to 100% exactly

### Neuropsychological assessment

Descriptive neuropsychological performances are provided per participant in Table 4. Regarding intellectual functions, and considering descriptive classifications proposed in the WASI-II manual, only 3/11 presented average level and 3/11 presented extremely low level. Regarding executive functions, 5/11 participants had impaired scores (< 16). An additional 3 participants presented limit scores (= 16). For the assessment of apathy, 5/11 participants were considered clinically apathetic according to the cut-off score of –16. When using the intensity of apathy’s classification, 3/11 participants were severely apathetic, 2/11 were moderately apathetic, 6/11 had a tendency to apathy, and none presented no apathy.

**Table 4:** Neuropsychological performances per participant (n = 11)*

Neuropsychological tests	Participant's no.											Mean (SD)
	1	2	3	4	5	6	7	8	9	10	11	
Wechsler Abbreviated Scale of Intelligence^Ϯ^												
Global score	64	93	81	65	72	75	73	65	82	93	92	77.7 (11.3)
Verbal intelligence quotient	64	90	73	67	59	67	84	73	98	76	87	76.2 (12.2)
Performance intelligence quotient	70	98	94	55	76	88	68	60	71	112	99	81.0 (18.2)
Frontal assessment battery (/18)	17	15*	16	14*	15*	14*	14*	16	16	18	17	
Lille Rating Apathy Scale (− 36 to + 36)	− 18	− 14*	− 6*	− 5*	− 20	− 18	− 5*	− 21	− 20	− 21	− 16*	

*Score under normative data

^Ϯ^No cut-off score but classification (see manuscript)

^1^Norms are different for males and females

### IADLs accomplishment and cognitive functioning

Results indicated that the only 3/11 participants that were independent in IADLs total score had normal scores in all cognitive batteries (Table 5). All participants except one who categorized as semi-independent had borderline level of intelligence and deficits in executive functions. Interestingly, the only dependent participant with average intellectual status had impaired executive functioning. Correlations between IADLs accomplishment sub-scores and cognitive scores are presented in Table 6. A moderate positive association was found between the WASI-II full-scale IQ score with home management and transportation.

**Table 5:** IQ and executive functions scores classified according to level of independence (n = 11)

Profile based on ILS	Participants no.	Full-scale IQ	Executive functions
Dependent	1	Extremely low	Average
	2	Average	Impaired
	3	Low average	Limit
	4	Extremely low	Impaired
Semi-independent	5	Borderline	Impaired
	6	Borderline	Impaired
	7	Borderline	Impaired
	8	Extremely low	Limit
Independent	9	Low average	Limit
	10	Average	Average
	11	Average	Average

**Table 6:** Spearman’s rho correlations between the ILS subscale scores and intellectual, executive and apathy scores (n = 11)

	WASI-II total score	FAB total score	LARS total score
	*r* correlation	*r* correlation	*r* correlation
Memory/orientation	.133	.867	− .611
Money management	.563	.215	− .508
Home management and transportation	.705	.153	− .388
Health and safety	.346	.135	− .423
Social adjustment	− .179	.296	.050
Subscale total sum	.498	.321	− .550

## Discussion

This exploratory study is the first step towards a better understanding of independent living and its links to cognitive functioning among adults who have grown with the childhood phenotype of DM1. This study brings some evidence of the large variety of difficulties encountered by this population in performing IADLs. Even more importantly, it highlights their need for support to accomplish these activities which are essential to function in everyday life. In addition, a large proportion of participants showed an intellectual functioning below average which is related to the level of dependency exhibited by the individuals. An evaluation of the IADLs should be recommended in this population, notably for individuals with lower IQs.

Considering that there is currently no published study addressing the ability to accomplish IADLs among adults with childhood phenotype of DM1, no comparison can be made with other data. However, results emerging from this research are in line with the retrospective study of Gagnon et al. (2017), which had raised the presence of participation restrictions among this population in domestic life, education and employment.

First of all, if growing up with DM1 as a child may promote anxiety and social withdrawal in children and even teenagers (11), it is to note that considering the low levels of state anxiety found in participants, anxiety seem not to contribute significantly in the difficulties observed in the accomplishment of the IADLs assessed in the study.

Money management was the activity with the highest proportion of dependent participants (8/11). Achieving money management tasks (paying bills, writing a check, planning a budget) involve a range of complex cognitive abilities (e.g., planning, calculation, working memory, problem solving, etc.). Working memory impairment (12) and, more globally, lower intelligence (9,10) have been previously documented in children with the childhood phenotype of DM1, which might contribute to participants’ lower level of independence in this category. These scores reflect problematic situations often reported by patients during clinical follow-up (i.e., bankruptcy, eviction, not paying rent in time, etc.). Health & safety activities is the category with the highest number of independent participants (6/11). This category assesses the individual’s awareness of personal health status and ability to evaluate health problems, handle medical emergencies, and take safety precautions. One of our hypotheses to explain relatively high scores in this category is that these abilities are developed early in and are reinforced throughout our life (i.e., learning not to talk to strangers, looking both sides before crossing the road, etc.). We suspect that these behaviors become habits that are more easily maintained with time, as opposed to more complex abilities such as money management. Moreover, the fact that participants from the Saguenay-Lac-Saint-Jean region are followed up by a nurse case manager during their lifetime, with whom they have a continuous and close relationship, may contribute to their good knowledge on appropriate behaviors in relation to health and security [33].

Health records showed that, for all IADLs, the percentage of participants who needed help from their family and/or from other external resources ranged from 8/11 to 10/11 (82 to 91%), suggesting that only few participants were able to perform by themselves the IADLs required for independent living. This is in accordance with the previous study of Gagnon et al., which revealed that the majority of individuals with this phenotype performed house-related responsibilities adequately, but with services or help [34].

Considering the total scores of the ILS, 8/11 participants were either dependent or semi-independent, meaning that a high proportion of patients experienced difficulties in their everyday life. Adults living with childhood phenotype of DM1 would thus benefit from interventions to help them maintain their ability to carry out IADLs. Moreover, considering the young age of the present sample and the fact that DM1 is a progressive disease, it is essential that health care providers keep in mind the evolution of those difficulties and their long-term impact on quality of life.

The home management and transportation subscale was associated to the global IQ, as reported in the ILS manual. This tends to support the idea that the ability to accomplish some IADLs may be influenced by intellectual functioning, although intelligence tests did not directly assess the ability of an individual to perform activities of daily living. Surprisingly, the executive performance scores were not correlated with the instrumental activities of the ILS involving executive functions such as money management. The FAB is a short questionnaire assessing different aspects of executive functions that might not be specific enough to evidence the cognitive functions involved in those ILS subscales. As an example, the manual of the ILS revealed that the money management subscale show a moderately high correlation with the arithmetic subtest of the WAIS-R [26], thus providing support that it requires specific cognitive functions that are not assessed in the FAB neither the WASI-II.

In a descriptive manner, patients presenting lower IQs, limit or impaired executive functions abilities showed difficulties in performing IADLs, thus raising the concern of the functional impact of those impairments. Accordingly, when neuropsychological clinical evaluation reveals an IQ in the limit range in a patient with the childhood phenotype of DM1, it should be referenced to an occupational therapist to validate abilities to perform IADLs.

The high rate of apathy observed in the current study (5/11 participants) may directly impact the level of activities performed by the individual and their need of help from relatives. Preserved motivation and self-activation are required to initiate, execute and maintain daily activities such as housekeeping and meal preparation. However, in the present study, apathy was not associated to IADLs accomplishment. Difficulties described in the current study might be underestimated considering that results from the ILS were mostly obtained through scenarios regarding IADLs instead of real-life tasks, therefore not considering tangible apathy and initiative abilities. Future studies should consider a more holistic approach, assessing individuals in their real-life context in order to understand more precisely where and when difficulties emerge.

As suggested by Van Heugten et al., it is imperative that rehabilitation services in DM1 address not only motor but also neuropsychological symptoms, especially in the childhood phenotype [35]. For example, acceptability and feasibility of Stanford Chronic Disease Self-Management Program has been previously assessed with patients with DM1 and presented considerable potential [36]. Moreover, as suggested by authors such as Fujino et al. or Graham et al. [37, 38] and considering the implication of cognitive area on IADLs found in this study, neuropsychological rehabilitation techniques could be useful to compensate for cognitive and behavioral impairments and improve patients’ abilities to perform independently IADLs; it could include, among others, goal management training and problem-solving training. Finally, neuropsychologists have an important role in risk reduction by helping patients and colleagues of the multidisciplinary teams understanding more precisely the problematic of cognitive impairments and helping them to notice how they impact their ability to perform IADLs.

### Limits

This study has some limits to be considered. First, this exploratory study was performed with a limited number of participants (n = 11), which restrict its generalization. Although those 11 participants were specifically chosen to be a representative sample of the population to limit bias, they can possibly represent the most active DM1 childhood individuals as they accepted to be part of the study. Also, tests assessing intellectual and executive functioning were purposely brief to avoid time and fatigue burden in participants, because the aim of the study was to assess both the performance in IADLs and cognition. A more comprehensive neuropsychological battery may be contributive in further studies.

## Conclusions

This study demonstrated that DM1 patients with the childhood phenotype experience difficulties with some specific IADLs. As shown with descriptive results, the cognitive profile of the participants is in line with their level of dependence in IADLs. This study enhances the importance of guiding patients through the continuum of services available from their young age to their adult life. They need to be supported and offered with all the help necessary to accomplish IADLs. Larger studies need to be conducted in order to establish a more detailed portrait of their difficulties in maintaining independent living and the links with neuropsychological impairments.

## Data Availability

The de-identified datasets used and analysed during the current study are available from the corresponding author on reasonable request.

## Abbreviations

ADL: Activity of daily living
BADL: Basic activity of daily living
CNS: Central nervous system
DM1: Myotonic dystrophy type 1
FAB: Frontal Assessment Battery
IADL: Instrumental activity of daily living
ILS: Independent Living Scale
IQ: Intellectual quotient
LARS: Lille Rating Apathy Scale
WASI-II: Wechsler Abbreviated Scale of Intelligence II
WAIS-R: Wechsler Adult Intelligence Scale—revised

## References

[1] Harper P (2001). Myotonic dystrophy.

[2] Hilton-Jones D, Turner MR (2014). Oxford textbook of neuromuscular disorders.

[3] Yum K, Wang ET, Kalsotra A (2017). Myotonic dystrophy: disease repeat range, penetrance, age of onset, and relationship between repeat size and phenotypes. Curr Opin Genet Dev.

[4] De Antonio M, Dogan C, Hamroun D, Mati M, Zerrouki S, Eymard B (2016). Unravelling the myotonic dystrophy type 1 clinical spectrum: a systematic registry-based study with implications for disease classification. Rev Neurol (Paris).

[5] Koch MC, Grimm T, Harley HG, Harper PS (1991). Genetic risks for children of women with myotonic dystrophy. Am J Hum Genet.

[6] Petitclerc E, Hebert LJ, Mathieu J, Desrosiers J, Gagnon C (2017). Lower limb muscle strength impairment in late-onset and adult myotonic dystrophy type 1 phenotypes. Muscle Nerve.

[7] Raymond K, Levasseur M, Mathieu J, Gagnon C (2019). Progressive decline in daily and social activities: a 9-year longitudinal study of participation in myotonic dystrophy type 1. Arch Phys Med Rehabil.

[8] Harper PS, Harley HG, Reardon W, Shaw DJ (1992). Anticipation in myotonic dystrophy: new light on an old problem. Am J Hum Genet.

[9] Angeard N, Gargiulo M, Jacquette A, Radvanyi H, Eymard B, Héron D (2007). Cognitive profile in childhood myotonic dystrophy type 1: is there a global impairment?. Neuromuscul Disord.

[10] Ekström AB, Hakenäs-Plate L, Tulinius L, Wentz E (2009). Cognition and adaptive skills in myotonic dystrophy type 1: a study of 55 individuals with congenital and childhood forms. Dev Med Child Neurol.

[11] Douniol M, Jacquette A, Cohen D, Bodeau N, Rachidi L, Angeard N (2012). Psychiatric and cognitive phenotype of childhood myotonic dystrophy type 1. Dev Med Child Neurol.

[12] Angeard N, Jacquette A, Gargiulo M, Radvanyi H, Moutier S, Eymard B (2011). A new window on neurocognitive dysfunction in the childhood form of myotonic dystrophy type 1 (DM1). Neuromuscul Disord.

[13] Echenne B, Rideau A, Roubertie A, Sébire G, Rivier F, Lemieux B (2008). Myotonic dystrophy type I in childhood Long-term evolution in patients surviving the neonatal period. Eur J Paediatr Neurol.

[14] Steyaert J, Umans S, Willekens D, Legius E, Pijkels E, de Die-Smulders C (1997). A study of the cognitive and psychological profile in 16 children with congenital or juvenile myotonic dystrophy. Clin Genet.

[15] Lindeblad G, Kroksmark AK, Ekström AB (2019). Cognitive and adaptive functioning in congenital and childhood forms of myotonic dystrophy type 1: a longitudinal study. Dev Med Child Neurol.

[16] Raymond K, Levasseur M, Mathieu J, Desrosiers J, Gagnon C (2017). A 9-year follow-up study of the natural progression of upper limb performance in myotonic dystrophy type 1: a similar decline for phenotypes but not for gender. Neuromuscul Disord.

[17] Laberge L, Mathieu J, Auclair J, Gagnon E, Noreau L, Gagnon C (2013). Clinical, psychosocial, and central correlates of quality of life in myotonic dystrophy type 1 patients. Eur Neurol.

[18] Pérès K, Helmer C, Amieva H, Orgogozo JM, Rouch I, Dartigues JF (2008). Natural history of decline in instrumental activities of daily living performance over the 10 years preceding the clinical diagnosis of dementia: a prospective population-based study. J Am Geriatr Soc.

[19] Jefferson AL, Cahn-Weiner D, Boyle P, Paul RH, Moser DJ, Gordon N (2006). Cognitive predictors of functional decline in vascular dementia. Int J Geriatr Psychiatry.

[20] Dodge HH, Du Y, Saxton JA, Ganguli M (2006). Cognitive domains and trajectories of functional independence in nondemented elderly persons. J Gerontol A Biol Sci Med Sci.

[21] Schmitter-Edgecombe M, Woo E, Greeley DR (2009). Characterizing multiple memory deficits and their relation to everyday functioning in individuals with mild cognitive impairment. Neuropsychology.

[22] Aretouli E, Brandt J (2010). Everyday functioning in mild cognitive impairment and its relationship with executive cognition. Int J Geriatr Psychiatry.

[23] Cahn-Weiner DA, Farias ST, Julian L, Harvey DJ, Kramer JH, Reed BR (2007). Cognitive and neuroimaging predictors of instrumental activities of daily living. J Int Neuropsychol Soc.

[24] Association AOT (2017). Occupational therapy practice framework: domain and process (3rd edition). Am J Occup Therapy.

[25] De Vriendt P, Gorus E, Cornelis E, Velghe A, Petrovic M, Mets T (2012). The process of decline in advanced activities of daily living: a qualitative explorative study in mild cognitive impairment. Int Psychogeriatr.

[26] Loeb PA (1996). Independent living scales manual.

[27] Wechsler D (2011). WASI-II: Wechsler abbreviated scale of intelligence.

[28] Dubois B, Slachevsky A, Litvan I, Pillon B (2000). The FAB: a frontal assessment battery at bedside. Neurology.

[29] Sockeel P, Dujardin K, Devos D, Deneve C, Destee A, Defebvre L (2006). The Lille apathy rating scale (LARS), a new instrument for detecting and quantifying apathy: validation in Parkinson's disease. J Neurol Neurosurg Psychiatry.

[30] Starkstein SE, Petracca G, Chemerinski E, Kremer J (2001). Syndromic validity of apathy in Alzheimer's disease. Am J Psychiatry.

[31] Lechowski L, Benoit M, Chassagne P, Vedel I, Tortrat D, Teillet L (2009). Persistent apathy in Alzheimer's disease as an independent factor of rapid functional decline: the REAL longitudinal cohort study. Int J Geriatr Psychiatry.

[32] Spielberger CD (1983). Manual for the State-trait anxiety inventory (form Y) ("self-evaluation questionnaire").

[33] Gagnon C, Chouinard M, Lavoie M, Champagne F (2010). Analyse du rôle de l'infirmière dans le suivi des personnes atteintes de maladies neuromusculaires. Can J Neurosci Nurs.

[34] Gagnon C, Kierkegaard M, Blackburn C, Chrestian N, Lavoie M, Bouchard MF (2017). Participation restriction in childhood phenotype of myotonic dystrophy type 1: a systematic retrospective chart review. Dev Med Child Neurol.

[35] Van Heugten C, Meuleman S, Hellebrekers D, Kruitwagen-van Reenen E, Visser-Meily J (2018). Participation and the role of neuropsychological functioning in myotonic dystrophy type 1. J Neuromuscul Dis.

[36] Raymond K, Levasseur M, Chouinard MC, Mathieu J, Gagnon C (2016). Stanford chronic disease self-management program in myotonic dystrophy: new opportunities for occupational therapists: Stanford Chronic Disease Self-Management Program dans la dystrophie myotonique : De nouvelles opportunites pour les ergotherapeutes. Can J Occup Ther.

[37] Fujino H, Shingaki H, Suwazono S, Ueda Y, Wada C, Nakayama T (2018). Cognitive impairment and quality of life in patients with myotonic dystrophy type 1. Muscle Nerve.

[38] Graham CD, Kemp S, Radakovic R, Kapur N (2018). Clinical neuropsychology in the management of myotonic dystrophy. Muscle Nerve.

